# Changes in Soil Microbial Community Structure Influenced by Agricultural Management Practices in a Mediterranean Agro-Ecosystem

**DOI:** 10.1371/journal.pone.0080522

**Published:** 2013-11-18

**Authors:** Fuensanta García-Orenes, Alicia Morugán-Coronado, Raul Zornoza, Kate Scow

**Affiliations:** 1 Department of Agrochemistry and Environment, University Miguel HernándezElche, Alicante, Spain; 2 Deparment of Geography, Universitat de València, València, Spain; 3 Department of Agrarian Science and Technology, Universidad Politécnica de Cartagena, Cartagena, Murcia, Spain; 4 Department of Land, Air and Water Resources, University of California Davis, Davis, California, United States of America; Cinvestav, Mexico

## Abstract

Agricultural practices have proven to be unsuitable in many cases, causing considerable reductions in soil quality. Land management practices can provide solutions to this problem and contribute to get a sustainable agriculture model. The main objective of this work was to assess the effect of different agricultural management practices on soil microbial community structure (evaluated as abundance of phospholipid fatty acids, PLFA). Five different treatments were selected, based on the most common practices used by farmers in the study area (eastern Spain): residual herbicides, tillage, tillage with oats and oats straw mulching; these agricultural practices were evaluated against an abandoned land after farming and an adjacent long term wild forest coverage. The results showed a substantial level of differentiation in the microbial community structure, in terms of management practices, which was highly associated with soil organic matter content. Addition of oats straw led to a microbial community structure closer to wild forest coverage soil, associated with increases in organic carbon, microbial biomass and fungal abundances. The microbial community composition of the abandoned agricultural soil was characterised by increases in both fungal abundances and the metabolic quotient (soil respiration per unit of microbial biomass), suggesting an increase in the stability of organic carbon. The ratio of bacteria:fungi was higher in wild forest coverage and land abandoned systems, as well as in the soil treated with oat straw. The most intensively managed soils showed higher abundances of bacteria and actinobacteria. Thus, the application of organic matter, such as oats straw, appears to be a sustainable management practice that enhances organic carbon, microbial biomass and activity and fungal abundances, thereby changing the microbial community structure to one more similar to those observed in soils under wild forest coverage.

## Introduction

Soils represent the most diverse and important ecosystem on the planet [1]. Most of the biodiversity of agroecosystems is found in the soil [2], and the functions performed by soil biota have considerable direct and indirect effects on crop growth and quality, nutrient cycle quality and the sustainability of soil productivity [1]. Soil biota also contributes substantially to the resistance and resilience of agroecosystems to abiotic disturbance and stress [3]. The microbial members of soil communities are the most sensitive and rapid indicators of perturbations and land use changes. In this sense, a quantitative description of microbial community structure and diversity has aroused great interest as a potential tool for soil quality evaluation [4,5].

Agricultural land management is one of most significant anthropogenic activities that greatly alters soil characteristics, including physical, chemical, and biological properties [6]. This fact is particularly relevant in Mediterranean environments, where unsuitable land management together with climatic constraints (scarce and irregular rainfall and frequent drought periods) can contribute to increased rates of erosion and other degradation processes of agricultural land [7]. These conditions can lead to a loss in soil fertility and a reduction in the abundance and diversity of soil microorganisms. Agricultural management influences soil microorganisms and soil microbial processes by changing the quantity and quality of plant residues entering the soil and their spatial distribution, through changes in nutrients and inputs [8]. The excessive use of pesticides can drastically modify the function and structure of soil microbial communities, thereby altering the normal functioning of terrestrial ecosystems, which in turn has important implications for soil quality [9]. Management practices have a direct effect on soil microbiota, and the direct seeding of extensive crops increases microbial biomass [10,11]. Soils subjected to disturbance by tillage, however, can be more susceptible to reductions in soil microbiota due to desiccation, mechanical destruction, soil compaction, reduce pore volume, and disruption of access to food resources [12]. Some organic fertilisers, such as manure and sewage sludge, promote the activities of soil microbial communities [13,14]; however, repeated application of manures may pose environmental hazards, as they introduce faecal microbial flora into soil and have the potential to alter the endogenous microbial structure [15]. To improve or maintain the soil quality and biodiversity, the development and implementation of new sustainable agricultural practices is necessary. Currently, several strategies are being tested on experimental farms to reduce the high erosion rates and improve soil quality. On rainfed agricultural lands in eastern Spain, these practices include catch crops, no-tillage or reduced tillage, chipped pruned branches, straw mulch and weed control by herbicides [16]. Previous studies (carried out at the site that is also the subject of the current work) have shown that several of these strategies contribute to reductions in soil erosion [17] and enhance both soil quality and aggregate stability [18]. It is very important to understand the impact of these sustainable agriculture management practices on the microorganism species diversity and structure. Such data are critical to evaluate the effect that these new strategies may have on microbial communities in the light of sustainable goals because microbial communities are crucial to maintaining the soil quality and to developing a sustainable agricultural model, based not only on crop productivity but also on ecological principles.

To measure changes in the soil microbial community, the phospholipid fatty acid (PLFA) analysis was used. This analysis uses the lipids of the microbial membranes as biomarkers for specific groups of microorganisms and also creates a profile or fingerprint of the community structure. As a consequence, rapid changes in soil microbial community structure can be detected by changes in the PLFA pattern [4]. In addition, the total concentration of PLFAs can be used as a measure of viable microbial biomass because phospholipids are rapidly degraded after cell death [19]. PLFA analysis has been used to assess changes in soil microbial community structure as a consequence of various perturbations or management practices [5,20–22]. 

Most studies on microbial soil community structure and composition in agricultural fields have been restricted to areas using conventional (tillage) management or conventional agricultural practices, such as inorganic fertilisation or the use of herbicides, and very few studies have been conducted under Mediterranean conditions. In this study, we hypothesised that different agricultural management practices can modify, to varying extents, the soil microbial community structure of a rainfed orchard. An adjacent area, which had never been cultivated and contained only wild vegetation coverage, was used as a standard for local, high quality soil to be compared with soil from the agricultural practices tested, in terms of microbial responses. We aimed to investigate: (i) the effects of different management practices on the soil microbial community, (ii) the relationship between soil physicochemical and biochemical properties and the microbial community structure, and (iii) the most sustainable management system to promote a more abundant microbial community.

## Results

### Soil physical, chemical and biochemical properties

All properties analysed were significantly higher in the soil treated with oat straw mulch compared to the other management systems, reaching similar values than the soil with wild forest coverage (Table 1). As a general pattern, most properties increased in terms of the management systems used as follows: RH ≈ T ≈ OT < LA < OS < WF. The C/N ration and qCO_2_ were not significantly different among treatments.

**Table 1:** Main physical, chemical and biochemical properties measured in soils for each treatment.

Management practices	AS^a^	Corg	N	C/N	WHC	P	Csol	BSR	Cmic	qCO_2_
RH	41±6	13.9±1.5a	1.1±0.1a	13.2±0.5	46.5±1.6a	1.75±0.46a	44.8±16.3a	0.52±0.20a	152±80a	3.94±1.34
T	46±7	14.5±0.3a	1.1±0.1a	13.3±1.3	45.5±1.0a	1.66±0.26a	51.8±9.6a	1.42±0.30b	299±50a	4.74±0.67
OT	50±7	14.4±0.9a	1.2±0.0a	13.3±0.2	46.8±7.3ab	1.64±0.20a	59.6±17.3a	0.75±0.05ab	180±83a	4.79±2.07
OS	79±9	27.2±4.1b	2.2±0.2b	12.8±0.7	54.4±1.3bc	3.63±0.34c	125.8±46.3c	2.35±0.37c	576±171c	3.85±1.38
C	66±4	18.2±1.8a	1.7±0.1a	13.4±0.3	48.9±1.5ab	1.62±0.14a	85.4±8.2b	1.81±0.29bc	329±70b	5.89±0.67
NC	84±7	31.6±3.3b	2.2±0.9b	13.8±1.2	56.0±4.1c	2.49±0.15b	93.8±28.6bc	2.40±0.13c	400±167bc	4.90±0.14
F-value^b^	40.2**	16.04**	27.87**	0.47 ns	7.60*	40.13**	21.74**	30.27**	16.08**	0.79 ns

Values are the mean ± standard deviation.

^a^ AS: aggregates stability (%); Corg: soil organic carbon (g kg^-1^); N: total Nitrogen (g k^- 1^); WHC: water holding capacity (%); P: Available Phosphorous (mg/kg), Csol: soluble organic carbon (mg kg^-1^); Cmic: microbial biomass carbon (mg C kg^-1^ soil); BSR: Basal soil respiration (mg C-CO_2_kg^-1^ h^-1^); qCO_2_: BSR/Cmic. ^b^Significant at: *P<0.05, **P<0.001; ns: not significant (*P*>0.05). Different letters indicate significant differences (*P*<0.05) among means.

### Multivariate analysis

**Figure 1:**
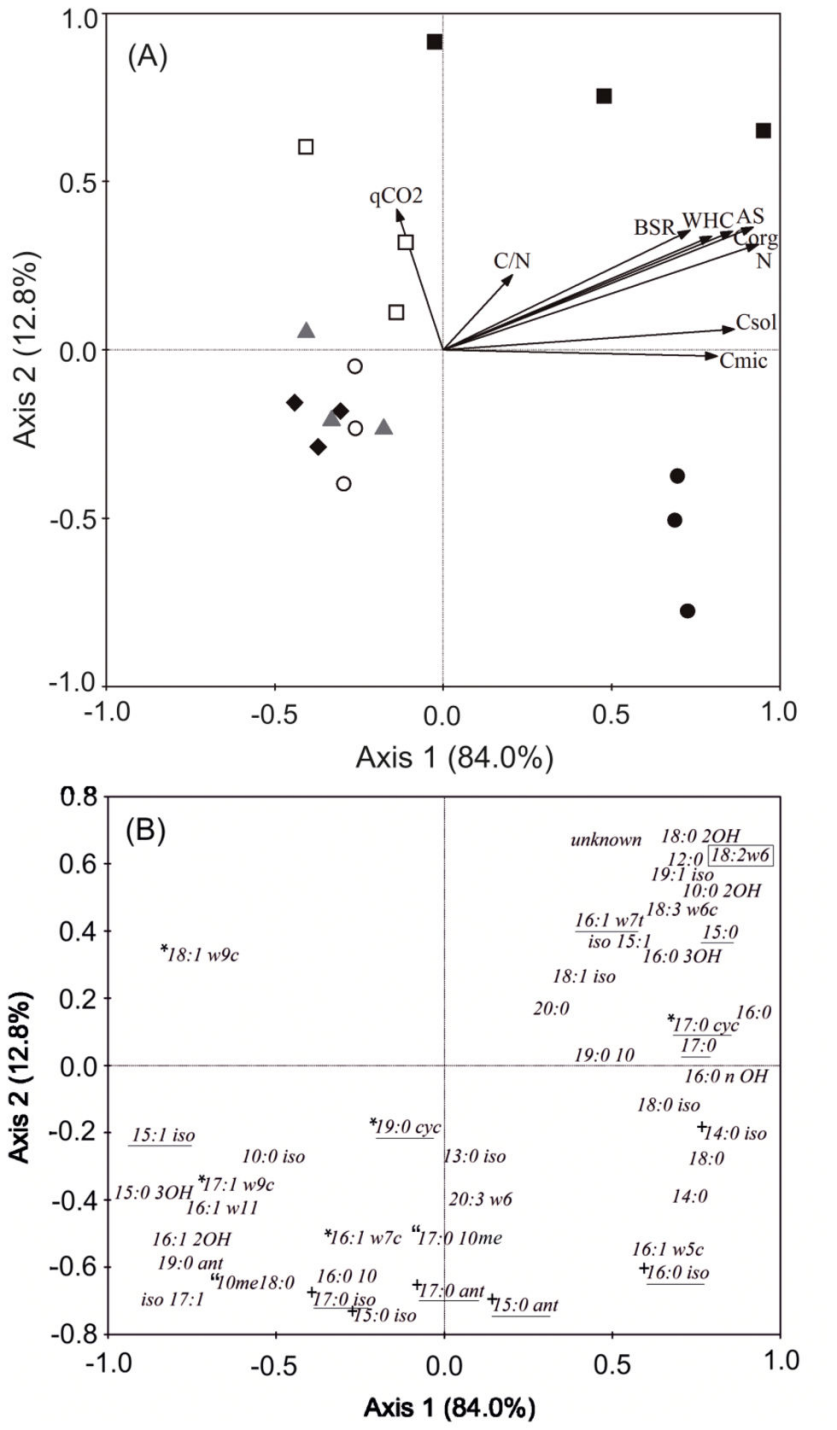
Samples and soil characteristics biplots (A) and loadings plots (B) from RDA performed on the relative concentration of PLFAs in all management practices:residual herbicide (♦), tillage (▲), oats+tillage (○), oats straw (●), land abandonment (□) and wild forest coverage (■). PLFAs used for microbial groups designation are marked as: underlined (bacteria), framed (fungi), * (G- bacteria), + (G+ bacteria) and “ (actinobacteria). Corg: soil organic carbon; N: total nitrogen; Csol: soluble carbon; WHC: water holding capacity; AS: aggregate stability; Cmic: microbial biomass C; BSR: basal soil respiration; qCO_2_: BSR/Cmic.

The RDA performed on all data (Figure 1) showed that the first two axes could explain 96.8% of the total variation. Axis 1 separated OS and WF from the other management practices and explained 84.0% of the variation, while axis 2, which separated WF from OS, and LA from RH, T and OT, explained 12.8% of the variation. All soil physical, chemical and biochemical properties, with the exception of available P, were significant (*P*<0.05) in explaining the variation in PLFA data (Figure 1A). The variables Corg, Csol, Cmic, N, AS, and BSR accounted for a large amount of the variation in the distribution of samples along axis 1, and thus with wild forest coverage and the oat straw management practice. Therefore, because all these variables depend on soil organic matter (SOM) content, SOM content largely accounts for variations in microbial community composition in terms of management practices. The qCO_2_ and the C/N ratio were significant in explaining the variation in microbial community composition along axis 2 (Monte Carlo, P<0.05; Figure 1A).The two axes of the RDA were significant in explaining the variation in the PLFAs (Figure 1B). PLFAs with a high score on an RDA axis are strongly related to the axis and to the environmental variable defining the axis [23]. WF soil had high concentrations of the saturated PLFAs 12:0, 10:0 2OH, 15:0, 16:0, 16:0 3OH, 20:0 and 18:0 2OH, the unsaturated PLFAs 15:1iso, 16:1ω7t, 18:1iso, and 19:1iso, the unsaturated PLFAs related to fungal biomass 18:2ω6 and 18:3ω6c, and an unknown fatty acid. The soil treated with oat straw was characterised by high concentrations of the fatty acid 16:1ω5c, which was proposed to be an indicator of vesicular-arbuscular mycorrhyzae (VAM) [24]; however, this suggestion has been disputed by Frostegård et al. [25], as this fatty acid has also been found in bacteria [26]. The unsaturated PLFAs 14:0, 14:0iso, 18:0, 18:0iso and 16:0iso were also high within the oat straw treatment. The land abandonment treatment had high concentrations of the unsaturated fatty acid related to G- bacteria biomass, 18:1ω9c. The remainder of the management practices (RH, T and OT) were clearly clustered and showed high concentrations of PLFAs that are mainly representative of bacteria, such as the saturated 15:0 3OH, 19:0, 10:0iso and 17:0iso, and the unsaturated 15:1iso, 17:1ω9c, 16:1ω11; 16:1 2OH and iso17:1; these soils were also enriched in the fatty acid 10me18:0, an indicator of actinobacteria. 

### PLFA biomarkers

**Figure 2:**
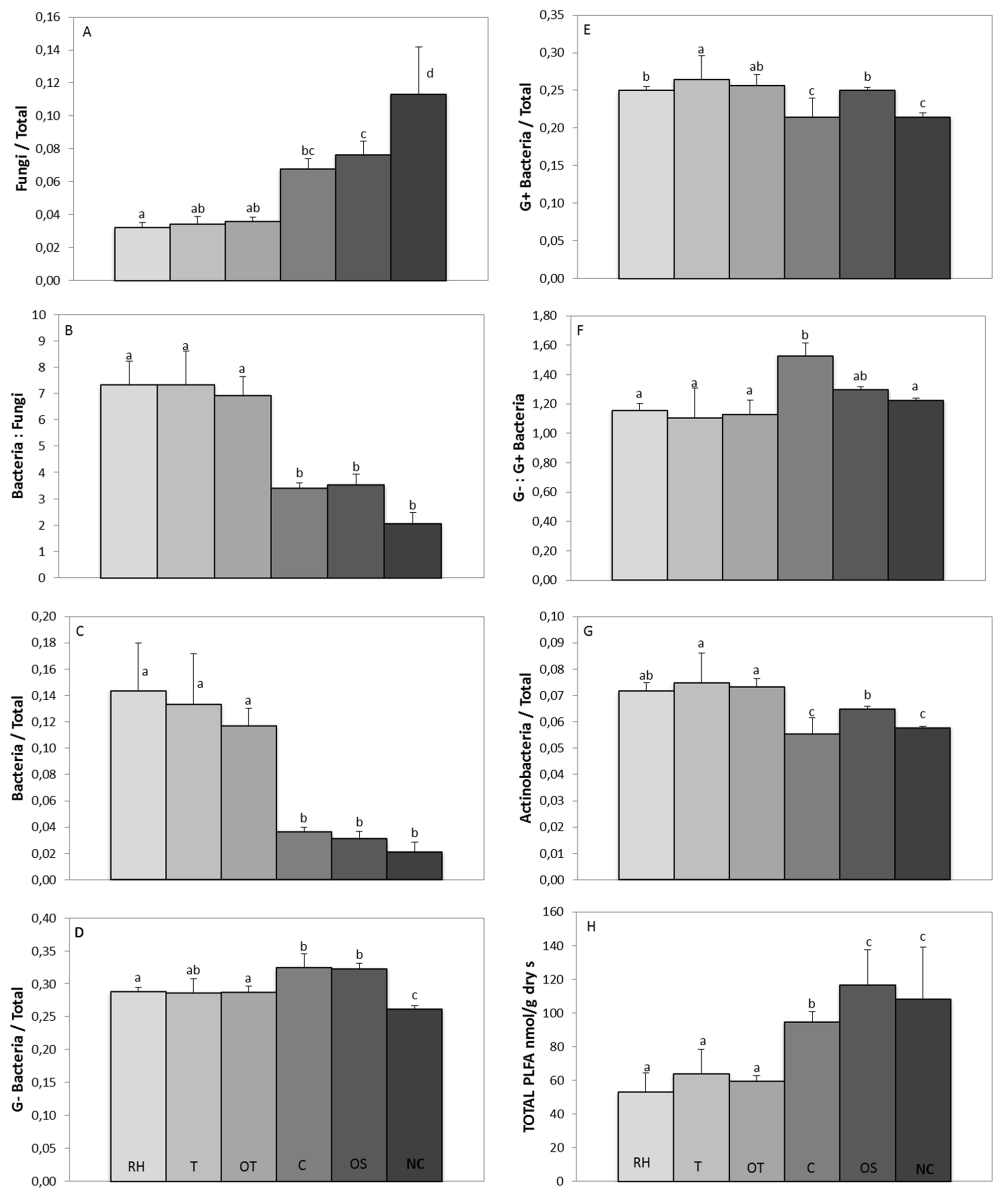
Values of total PLFAs biomass and various PLFA biomarkers (mean ± standard deviation) for all soil management practices. A one-way ANOVA and Tukey test (P<0.05) were used to compare significant differences. Different letters above the bars indicate significant differences among management practices. G+: Gram positive; G-: Gram-negative.

Figure 2 shows the specific PLFAs measured as biomarkers and total biomass. Measures of microbial biomass, estimated based on the total PLFA biomass (Figure 2H), indicated that the highest biomass was found in soils receiving oat straw amendment; no significance differences in biomass were observed between these soils and the soil with wild forest coverage. The rest of the treatments showed the following trend for the total PLFA content: Land abandonment> Tillage > Oat Tillage > Residual Herbicide, but only the land abandonment treatment was significantly different from the others. The proportion attributable to fungi was significantly higher (P< 0.05) in the soil used as a reference with wild forest coverage, with a general decreasing trend, as follows: WF>OS>LA>OT=T=RH. The relative abundance of bacteria, however, was significantly higher in the RH, T and OT treatments, with the lowest values observed in LA, OS and WF; no significant differences were found among these latter treatments. The ratio of bacteria:fungi followed the same pattern as that of relative total bacteria. As a general pattern, the relative abundances of G+ bacteria and actinobacteria were lower in LA and WF and higher in P and PO. The relative abundance of G- bacteria was higher in LA and OS. There were no great differences among management practices, in terms of the ratio of G-:G+ bacteria; however, significantly higher values were observed in LA. 

Table 2 shows the correlation coefficients between the different soil properties and the PLFA biomarkers. Total PLFA was strongly positively correlated with all soil properties, with the exception of the C/N ratio. The strongest correlation was found for N (r=0.92; P<0.01), followed by Corg and AS (r=0.88; P<0.01). In general, all biomarkers were positively correlated with the soil properties, except for the ratio of bacteria:fungi, which was negatively correlated with all properties except C/N.

**Table 2:** Correlation coefficients (r-values) for relationships between physical, chemical and biochemical properties and PLFA biomarkers.

	Corg	N	C/N	Csol	WHC	AS	P	Cmic	BSR	qCO_2_
Total PLFA	0.88^**^	0.92^**^	-0.059	0.83^**^	0.80^**^	0.88^**^	0.73^**^	0.80^**^	0.68^**^	0.07
Fungi	0.97^**^	0.94^**^	0.239	0.71^**^	0.81^**^	0.87^**^	0.59^**^	0.61^**^	0.64^**^	0.12
Bacteria:Fungi	-0.74^**^	-0.75^**^	-0.049	-0.69^**^	-0.70^**^	-0.88^**^	-0.52^*^	-0.53^*^	-0.76^**^	-0.35
Bacteria	0.85^**^	0.91^**^	-0.141	0.85^**^	0.77^**^	0.86^**^	0.82^**^	0.85^**^	0.67^**^	0.04
G-	0.77^**^	0.84^**^	-0.197	0.84^**^	0.71^**^	0.82^**^	0.74^**^	0.79^**^	0.71^**^	0.12
G+	0.85^**^	0.92^**^	-0.152	0.84^**^	0.77^**^	0.84^**^	0.83^**^	0.85^**^	0.65^**^	0.02
G-:G+	0.20	0.24	-0.207	0.37	0.22	0.36	0.11	0.23	0.51^*^	0.34
Actinobacteria	0.86^**^	0.92^**^	-0.135	0.83^**^	0.77^**^	0.83^**^	0.83^**^	0.85^**^	0.60^**^	-0.02

Corg: soil organic carbon; N: total Nitrogen; Csol: soluble carbon; WHC: water holding capacity; AS: aggregate stability; Cmic: microbial biomass carbon; BSR: basal soil respiration; P: available phosphorus; G-: Gram-negative bacteria; G+: Gram-positive bacteria; * and ** indicate significant correlation at P<0.05 and P<0.01, respectively.

## Discussion

The application of oat straw to soil has increased the organic carbon content and, as a consequence, there is an important increase of the microbial biomass. The total PLFAs are highly correlated with the microbial biomass carbon, determined by the fumigation-extraction method. The correlation obtained in this research between these parameters (r=0.80) is similar to that reported in previous works [5,27]. Our results suggest a substantial level of differentiation in the microbial community structure, according to the management practices assessed, as has also been observed by other authors [28–30]. These results corroborate the notion that the microbial community structure is a good indicator of soil quality, perturbations and the effects of different management practices [5,28], because the microorganisms respond against changes in soil management more rapidly than chemical or physical soil properties. 

The selected soil under wild forest coverage, not disturbed for long time, is used as high quality soil for reference, based on the fact that soils under wild and native vegetation that develop freely reach an equilibrium amongst their properties that leads to long-term stability [31]. The rest of treatments have been compared with this forest soil to assess which one supports a microbial community more similar to non-disturbed ecosystems, reference of high soil quality, since soil quality has been defined as “the capacity of a soil to function, within the limits imposed by the ecosystem, to preserve the biological productivity and environmental quality, and promote plant, animal and human health” [32]. Since the WF system supports a more abundant and richer microbial community, those agroecosystems with more abundant and diverse community, are supposed to be indicative of increased soil quality [5]. Increments in richness and biomass have been proposed as a basic objective in the management of degraded ecosystems [33,34]. Multivariate analysis showed that the microbial community structures of the soil with wild forest coverage and the soil receiving oat straw treatment were influenced by soil organic matter content. Microorganisms likely responded to increases in organic matter levels in the soil, involving increases in carbon, aggregate stability, increases in the capacity for water retention and higher microbial activity. It is important to note that the microbial community structure in the OS treatment falls within the positive range of axis 1 in the RDA, similar to the soil under wild forest coverage. Thus, the application of organic residues is a suitable management practice that enhances organic matter, improves structure and increases microbial biomass and activity in soils [16,35]. This practice also shifts soil microbial communities [36] to respond to increases in organic carbon. The increment of fungal proportions is directly related to the microbial community composition in OS and WF soils, as fungal communities play a dominant role in fresh organic matter decomposition [37]. The soil developed under wild forest coverage exhibited a community structure with higher values for both the C/N ratio and the metabolic quotient. This result may indicate a higher degree of stabilisation of soil organic matter, and microbial communities in this soil may need to use more recalcitrant carbon pools, compared to communities in the soil where oat straw was added, to remain at a steady state [38].

By contrast, the rest of the agricultural management practices shared a similar microbial community composition, bound to low contents of organic matter and characterised by low microbial size and activity and high proportions of bacteria and actinobacteria [5]. found that the microbial community structure of abandoned agricultural lands was not explained by any physical or chemical soil property measured, suggesting that the abandonment of the perturbation caused by agricultural activity per se and the associated changes in vegetation rather than direct changes in soil properties led to shifts in the soil microbial community structure. In this study, we found that the qCO_2_ largely accounts for variation in LA soil microbial community structure, which may be related to differences in the quality of organic matter. Organic matter is more stabilised in LA than in the soils receiving other treatments, so microbial communities need to use more recalcitrant carbon pools to remain at a steady state. The high concentration of the fungal PLFA 18:1w9c within the LA system supports this hypothesis, as fungi use more recalcitrant sources of C than bacteria [39,40].

Non-till management practices and the application of organic residues such as oat straw cause a consistent increase in the populations of fungi and Gram negative bacteria, and a significant decrease in the ratio of bacteria:fungi, as shown in previous reports [10,41–43]. The relatively high fungi abundance in OS and LA plots, compared to the other treatments, renders the soils under these management practices more similar to the soil with wild forest coverage, as previously reported [44–47]. Increments in the proportion of fungi are indicative of sustainable management practices since fungi are more sensitive to bacteria to perturbation [5,44]. Apart from increases in organic matter, the lack of tillage has also likely contributed to increases in fungal populations because tillage promotes the breakage of hyphae [48]. Bacteria are more abundant in the soils treated with herbicide and tillage, as they tend to dominate the decomposition and nutrient cycles in soils that are intensively managed, such as tilled, chemically fertilized, pesticides used, crop rotation, etc, [49–51]. The addition of herbicides can have toxic effects on microbial populations, thereby limiting the development of microorganisms [52]. reported that repeated residual herbicide application significantly decreased microbial biomass carbon and fungal biomass, as shown in our results. 

## Materials and Methods

### Ethics statement

No specific permits were required for the described field studies since these locations are not privately-owned or protected in any way. Field studies did not involve endangered or protected species.

### Study site

The experiment was established on a homogeneous terrain with a 5% slope at El Teularet Experimental Station [16] in the Enguera range (38°50’N; 0°42’W) in the southern part of the province of Valencia (eastern Spain). The soil is a Typic Xerorthent [53], with loam texture, developed from Cretaceous marls. The main soil characteristics are described in Table 3. The climate is typically Mediterranean, with 3-5 months of summer drought, usually from June to September. Mean annual rainfall at the study area is 479 mm, with an average annual temperature of 14.2 °C over the last ten years.

**Table 3:** Main characteristics of the study soil (0-5 cm depth).

Texture (% sand, silt, clay)^a^	39, 38, 23
pH (extract 1:5,w/v)	8.3 ± 0.02
Electrical conductivity (1:5, μS cm^-1^)	185 ± 4.00
CaCO_3_ (%)	60 ± 3.00
Total organic carbon (g kg^-1^)	12.5 ± 0.10
Soluble C (mg kg^-1^)	74 ± 1.00
Microbial biomass carbon (mg kg^-1^)	270 ± 2.00
Total N (g kg^-1^)	0.78 ± 0.03
Available P (mg kg^-1^)	2.00 ± 0.01
Extractable K (mg kg^-1^)	303 ± 12.00
Basal respiration rate (mg C-CO_2_ kg^-1^ h^-1^)	1.55 ± 0.08

Values are the mean ± standard deviation (n=20).

a Sand: 2-0.02 mm, Silt: 0.02-0.002 mm, Clay: <0.002 mm.

### Experimental design

The experimental area was ploughed to create uniform surface soil conditions prior to conducting our experiment. The area was then divided, and five different treatments were applied in February of 2004, with three replicate plots per treatment (6x10 m^2^) (Table 4). An adjacent area with the same type of soil under wild forest vegetation (WF) was used as a standard for local, high-quality soil. We used a soil under wild and native vegetation, as close as possible to potential vegetation [54], which had not been recently altered by human action (>60 years). The idea of using non disturbed soils under wild native vegetation was suggested by Fedoroff 1987 [55], and is based on the fact that soils that develop freely reach an equilibrium amongst their properties that leads to long-term stability, being considered as high quality soils [31]. The treatments were selected based on the practices commonly used by farmers in the study site, where rainfed agriculture is the most common farming activity (olive, almond and cereal crops). The treatments applied were as follows: residual herbicide (RH), tillage (T), oats and tillage (OT), oat straw (OS) and land abandonment (LA), where land was abandoned after farming, a common occurrence in recent decades in the agricultural areas of the Mediterranean basin.

**Table 4:** Description of the different soil agricultural management practices.

**Code**	**Treatments**	**Description**
RH	Residual herbicide	3 applications per year of oxyfluorfen (240 g L^-1^); 1.5 kg ha^-1^
T	Tillage	4 times per year (tillage depth: 20 cm)
OT	Oats + Tillage	Tillage: 4 times per year; (tillage depth: 20 cm) Sown 100% oats (ground and added to soil in spring)
LA	Land abandonment	Abandoned field with natural plant recolonisation (*Brachypodium retusum., Cistus albidus, Moricandia arvensis, Plantago lanceolata* and *Diplotaxis muralis*)
OS	Oat straw	Amount: 0.25 kg m^-2^ yr^-1^, straw mulch weed chopped add on surface of the soil.
WF	Wild forest coverage	Adjacent non-cultivated area (*Rhamnus lycioides, Quercus coccifera, Juniperus oxycedrus, Juniperu sphoenicea, Arbutus unedo, Chamaerops humilis, Lavandula latifolia, Lavandula dentata, Rosmarinus officinalis, Salvia blancoana, Thymus vulgaris, Erica multiflora* and *Cistus albidus*)**.**

### Soil sampling

Soil samples were collected in July of 2012. For each plot, six bulked sub-samples (100 cm^3^ cores) were randomly collected at 0-5 cm depth, and these sub-samples were homogenised to give one composite sample per plot. Field-moist soil samples were sieved at <2 mm and stored at the environmental temperature for physicochemical analysis. An aliquot of each soil sample was kept cool (4 °C) before the PLFA analysis. Soil sample aliquots were sieved between 0.25-4 mm to determine the percentage of stable aggregates. 

### Analytical methods

Soil organic carbon (Corg) was determined using the potassium dichromate oxidation method [56]. Aggregate stability (AS) was measured according to Roldán et al. [57], based on a prior study by Benito et al. [58]. This method examines the proportion of aggregates that remain stable after a soil sample is subjected to an artificial rainfall of known energy (279 J m^-2^). Total nitrogen (N) was determined by the Kjeldahl method [59]. Available phosphorus (P) was determined by the Burriel-Hernando method [60]. Water holding capacity (WHC) was assayed using the method proposed by Foster [61]. The microbial biomass carbon (Cmic) was determined by fumigation-extraction method [62], and the 0.5 M K_2_SO_4_ extracted carbon from non-fumigated samples was measured as soluble carbon (Csol). The basal soil respiration (BSR) was measured using a multiple sensor respirometer (Micro-Oxymax, Columbus, OH, USA). The qCO_2_ was calculated using the BSR/Cmic relationship.

Phospholipid fatty acid (PLFA) analysis was performed as described by Bossio et al. [28]. Briefly, fatty acids were extracted from 8 g of soil samples using a chloroform:methanol: phosphate buffer. PLFAs were separated from neutral and glycolipid fatty acids on a solid phase extraction column (0.58 Si; Supelco Inc., Bellafonte, PA, USA). After mild alkaline methanolysis, samples were analysed using a Hewlett Packard 6890 Gas Chromatograph with a 25 m Ultra 2 (5% phenyl)-methylpolysiloxane column (J and W Scientific, Folsom, CA, USA). Fatty acids were quantified by comparison of the peak areas with those of an internal standard 19:0 peak. The peaks were assigned using bacterial standards and identification software from the Microbial Identification System (Microbial ID, Inc., Newark, DE, USA). We identified 48 fatty acids. The fatty acid nomenclature used was described by Frostegård et al. [24]. PLFA were grouped into bacterial, fungal, Gram-negative [G−] bacteria, Gram-positive [G+] bacteria and actinobacteria following [63–65]. A detailed grouping is given in Table 5. Two ratios were also calculated: fungal to bacterial PLFAs (bacteria:fungi) and Gram-positive: Gram-negative bacteria (G-:G+ bacteria). The total biomass was estimated as the sum of all the extracted PLFAs (total PLFAs).

**Table 5:** Biomarker indices investigated.

**Group designation**	**Biomarkers**
Bacteria	i15:0, 15:0, a15:0, i16:0, 16:1ω7, i17:0, a17:0, cy17:0, 17:0, cy19:0
Fungi	18:2ω6
G- bacteria	cy17:0, cy19:0, 17:1ω9c, 16:1ω7c, 18:1ω9c
G+ bacteria	i14:0, i15:0, a15:0, i16:0, i17:0, a17:0
Actinobacteria	10Me17:0, 10Me18:0

### Statistical analyses

PLFA data were converted to mol% of the total fatty acid concentration. The fit of the data to a normal distribution for all properties measured was verified with the Kolmogorov-Smirnov test at P<0.05. Redundancy analysis (RDA) was used to examine the relationship between the microbial community composition and soil characteristics. Samples with similar PLFA profiles have similar scores and will therefore group closer together when plotted. Soil physical, chemical and biochemical properties were tested for significant contributions to the variation in the PLFA data using the Monte Carlo permutation test (P<0.05). Only the soil properties that were significantly correlated with factors in the RDA were included in the plots. Soil properties are represented by vectors. Vectors of greater magnitude that form smaller angles with an axis are more strongly correlated with that axis. RDA can be influenced by rare fatty acids. Fatty acids that only appear in a few samples are usually unreliably represented, as their values are near the detection limit. Hence, fatty acids that were present in less than 25% of the samples were omitted to avoid this problem at developing the analysis, taking out fatty acids that are unreliable represented. These fatty acids in the current study were: 13:0, a14:0, 18:1ω7 and 20:4ω6,9,12,15, which were present in <3 samples at very low concentrations.

We performed a one-way ANOVA to investigate the differences between management systems regarding microbial groups and ratios based on PLFA biomarkers, and comparisons between the means were made using the least significant difference (LSD) test, calculated at P<0.05. Pearson’s correlation coefficients (r) were calculated to assess the relationship between soil physicochemical and biochemical properties and PLFA microbial groups. 

RDA was performed using CANOCO for Windows, Version 4.54, while ANOVA and correlations were performed with the SPSS software for Windows, Version 18.0.

## Conclusions

Soil organic matter content seems to be the main agent responsible for the different microbial community structure under the different soil management practices. The most intensively managed soils had the highest relative abundance of bacteria and actinobacteria. The abandonment of agriculture has led to increase in microbial biomass and shifts in the microbial community structure, most likely due to the cessation of tillage, increases in organic matter and changes in organic matter quality, which led to increase in fungal populations. The application of oat straw increased the organic carbon content, microbial biomass and fungal populations, with a microbial community structure close to the soil used as reference under wild forest coverage. Thus, this study shows that the application of organic residues such as oat straw could represent an adequate solution for the sustainable maintenance of agricultural soils under Mediterranean conditions. Nonetheless, with the employed PLFA analysis is difficult to conclude if this management system is also supporting a healthy soil microbial community suitable to maintain healthy crops; thus, further research using other techniques with taxonomic resolution, such as DNA or RNA profiling, should be carried out to fill this gap. 
