# Correction: Changes in Soil Microbial Community Structure Influenced by Agricultural Management Practices in a Mediterranean Agro-Ecosystem

**DOI:** 10.1371/journal.pone.0152958

**Published:** 2016-03-31

**Authors:** Fuensanta García-Orenes, Alicia Morugán-Coronado, Raul Zornoza, Artemi Cerdà, Kate Scow

Dr. Artemi Cerdà should be included in the author byline instead of the Acknowledgements. He should be listed as the fourth author, and his affiliation is: **5** SEDER (Soil Erosion and Degradation Research Group), Department of Geography, Universitat de València, Blasco Ibañez, 28, 46010, València, Spain. The contributions of this author are as follows: Conceived and designed the experiments: AC.

The correct citation is: García-Orenes F, Morugán-Coronado A, Zornoza R, Cerdà A, Scow K (2013) Changes in Soil Microbial Community Structure Influenced by Agricultural Management Practices in a Mediterranean Agro-Ecosystem. PLoS ONE 8(11): e80522. doi:10.1371/journal.pone.0080522
